# Very low energy diets prior to bariatric surgery may reduce postoperative morbidity: a systematic review and meta-analysis of randomized controlled trials

**DOI:** 10.3389/fnut.2023.1211575

**Published:** 2023-06-20

**Authors:** Tyler McKechnie, Yung Lee, Joanna Dionne, Aristithes Doumouras, Sameer Parpia, Mohit Bhandari, Cagla Eskicioglu

**Affiliations:** ^1^Division of General Surgery, Department of Surgery, McMaster University, Hamilton, ON, Canada; ^2^Department of Health Research Methods, Evidence, and Impact, McMaster University, Hamilton, ON, Canada; ^3^Harvard T.H. Chan School of Public Health, Harvard University, Boston, MA, United States; ^4^Division of General Surgery, Department of Surgery, St. Joseph Healthcare, Hamilton, ON, Canada; ^5^Division of Orthopedic Surgery, Department of Surgery, McMaster University, Hamilton, ON, Canada

**Keywords:** bariatric surgery, weight loss, very low energy diet, very low calorie diet, postoperative complications, randomized controlled trials, systematic review

## Abstract

**Purpose:**

To optimize patients prior to bariatric surgery, very low energy diets (VLEDs) are often employed for 2–4 weeks preoperatively. They are known to result in preoperative weight loss, decrease liver volume, and decrease surgeon-perceived operative difficulty. Their impact on postoperative morbidity has been less extensively studied. We performed a focused systematic review and meta-analysis with the aim of comparing preoperative VLEDs prior to bariatric surgery with controls in terms of overall postoperative morbidity.

**Methods:**

MEDLINE, Embase, and CENTRAL were searched from database inception to February 2023. Articles were eligible for inclusion if they were randomized controlled trials (RCTs) comparing postoperative morbidity in adult patients (i.e., over the age of 18) receiving a VLED with liquid formulation to those receiving a non-VLED control prior to elective bariatric surgery. Outcomes included overall 30-day postoperative morbidity and preoperative weight loss. An inverse variance meta-analysis was performed with GRADE assessment of the quality of evidence.

**Results:**

After reviewing 2,525 citations, four RCTs with 294 patients receiving preoperative VLEDs with liquid formulation and 294 patients receiving a non-VLED control met inclusion. Patients receiving VLED experienced significantly more preoperative weight loss than patients receiving control (mean difference (MD) 3.38 kg, 95% confidence interval (CI) 1.06–5.70, *p* = 0.004, I^2^ = 95%). According to low certainty evidence, there was a non-significant reduction in 30-day postoperative morbidity in patients receiving VLED prior to bariatric surgery (risk ratio (RR) 0.67, 95%CI 0.39–1.17, *p* = 0.16, I^2^ = 0%).

**Conclusion:**

The impact of preoperative VLEDs on postoperative outcomes following bariatric surgery remains unclear. It is possible that VLEDs may contribute to decreased postoperative morbidity, but further larger prospective trials are required to investigate the signal identified in this study.

## Introduction

Obesity is a worldwide epidemic. More than 10% of the world population qualifies as obese, and this proportion is only expected to increase (1). Along with the rising prevalence of obesity, we have witnessed an explosion of weight reduction interventions. Among the most popular and effective to date is bariatric surgery (2). Bariatric surgery is widely regarded as the most sustainable form of weight loss and can improve several obesity-related comorbidities, such as type II diabetes, obstructive sleep apnea, and non-alcoholic fatty liver disease (3–5).

To optimize patients prior to bariatric surgery, very low energy diets (VLEDs) are often employed for 2–4 weeks preoperatively. Current Canadian Adult Obesity Clinical Practice Guidelines recommend 2–3 weeks of preoperative VLED aiming for 650–900 kilocalories (kcal) consumed daily with the use of commercially available liquid supplements such as Optifast^Ⓡ^ and Modifast^Ⓡ^ (6). Similarly, enhanced recovery after surgery (ERAS) guidelines for bariatric surgery recommend a 2–4-week period of VLED consumption (7). Prior randomized controlled trials (RCTs) evaluating VLEDs in bariatric surgery have shown effective preoperative weight loss with these interventions, as well as significant reductions in liver volume and visceral fat volume, with corresponding decreases in surgeon-perceived intraoperative difficulty (8–11). However, their impact on postoperative morbidity has been less extensively studied.

One of the earliest RCTs performed by Van Nieuwenhove et al. demonstrated a significant reduction in overall 30-day postoperative morbidity in patients receiving preoperative VLEDs prior to bariatric surgery (8). These results have not been reliably reproduced by subsequent RCTs (12, 13). A systematic review and meta-analysis in 2011 by Cassie et al. reported a significant reduction in postoperative complications in bariatric surgery patients receiving preoperative weight loss interventions compared with controls, but they combined randomized and non-randomized data (14). Additionally, multiple RCTs have been published. Contemporary systematic reviews have focused on reductions in liver volume and preoperative weight loss, with no meta-analyzing of postoperative morbidity data (15–17). As such, we performed a focused systematic review and meta-analysis with the aim of comparing preoperative VLEDs prior to bariatric surgery with controls in terms of overall postoperative morbidity. We hypothesize that preoperative VLEDs will induce significant weight reduction and associated with reduced postoperative morbidity as compared with control interventions.

## Materials and methods

### Search strategy

The following databases covering the period from database inception through February 2023 were searched: Medline, EMBASE, and Cochrane Central Register of Controlled Trials (CENTRAL). The search was designed and conducted by a medical research librarian with input from study investigators. Search terms included “bariatric surgery”, “gastric bypass”, “very low energy diet”, and “very low calorie diet” (complete search strategy available in Supplementary Tables 1, 2). The references of studies meeting inclusion criteria were searched manually to ensure that all relevant articles were included. This systematic review and meta-analysis are reported in accordance with the Preferred Reporting Items for Systematic Reviews and Meta-Analyses (PRISMA). The study protocol was registered on the International Prospective Register of Systematic Reviews (PROSPERO) *a priori* (CRD 42023403021; https://www.crd.york.ac.uk/prospero/display_record.php?RecordID=403021).

### Study selection

Articles were eligible for inclusion if they were randomized controlled trials (RCTs) comparing postoperative morbidity in adult patients (i.e., over the age of 18) receiving a VLED with liquid formulation to those receiving a non-VLED control prior to elective bariatric surgery. Only studies evaluating VLEDs with liquid formulation (e.g., Optifast^Ⓡ^, Modifast^Ⓡ^) were considered for inclusion to assess the impact of liquid formulation-based protocols on preoperative weight loss and postoperative morbidity. Studies evaluating lifestyle-based interventions (e.g., dieting, exercise) were not considered for inclusion. Studies in which VLEDs with liquid formulation were used in both the intervention and control groups were excluded. Any reported postoperative morbidity (i.e., overall postoperative morbidity, infectious morbidity, and wound complications) was considered adequate for inclusion. Studies that did not report postoperative morbidity were excluded. Single-armed studies evaluating VLEDs or comparative studies comparing two different types of VLEDs were not considered for inclusion. Finally, non-randomized studies, systematic reviews, meta-analyses, and editorials were excluded.

### Outcomes assessed

The outcomes were overall 30-day postoperative morbidity and preoperative weight loss in kilograms (kg). The majority of studies evaluating preoperative VLEDs with liquid formulation prior to bariatric surgery do not clearly define postoperative morbidity as it is not a commonly reported outcome and has yet to be analyzed as a primary outcome (8, 10, 12, 13, 18, 19). For the purposes of this review, postoperative morbidity was defined as any deviation from the expected postoperative course within 30 days of the index operation as reported by each included study. If studies reported overall morbidity as a pooled outcome, this was extracted preferentially, followed by overall infectious morbidity, gastrointestinal morbidity, and wound complications.

### Data extraction

Two reviewers independently evaluated the systematically searched titles and abstracts using a standardized, pilot-tested form. Discrepancies that occurred at the title and abstract screening phases were resolved by the inclusion of the study. At the full-text screening stage, discrepancies were resolved by consensus between the reviewers. If disagreement persisted, an additional reviewer was consulted. Two reviewers independently conducted data extraction into a data collection form designed *a priori*. The extracted data included study characteristics (e.g., author, year of publication, and study design), patient demographics (e.g., age, gender, body mass index [BMI], and comorbidities), treatment characteristics (e.g., VLED details, type of bariatric surgery), and postoperative morbidity (e.g., overall, infectious, wound).

### Risk of bias assessment and certainty of evidence

The risk of bias for RCTs was assessed using the Cochrane Risk of Bias Tool for Randomized Controlled Trials 2.0 (20). Quality of evidence for estimates derived from meta-analyses was assessed by Grading of Recommendations, Assessment, Development, and Evaluation (GRADE) (21). Two reviewers assessed the risk of bias and certainty of evidence independently. Discrepancies were discussed among the reviewers until a consensus was reached.

### Statistical analysis

All statistical analyses were performed on STATA version 15 (StataCorp, College, TX) and Cochrane Review Manager 5.3 (London, United Kingdom). A meta-analysis was performed using an inverse variance random effects model for all comparative data. Pooled effect estimates for binary outcomes were estimated with risk ratios (RR) along with their respective 95% confidence intervals (CI). Pooled effect estimates for continuous outcomes were estimated with mean differences (MD) along with their respective 95% CI. Mean and standard deviation (SD) were estimated for studies that only reported median and interquartile range (IQR) using the method described by Wan et al. (22). Missing SD data were, then, calculated according to the prognostic method (23). Assessment of the between-study heterogeneity was carried out using the I^2^ statistic. An I^2^ greater than 40% was considered to represent considerable heterogeneity (24). Bias in meta-analyzed outcomes was assessed with funnel plots when data from more than 10 studies were included in the analysis (25). A leave-one-out sensitivity analysis was performed by iteratively removing one study at a time from the inverse variance random effects model to ensure that pooled effect estimates were not driven by a single study. A systematic narrative summary was provided for each outcome.

## Results

### Study characteristics

**Figure 1:**
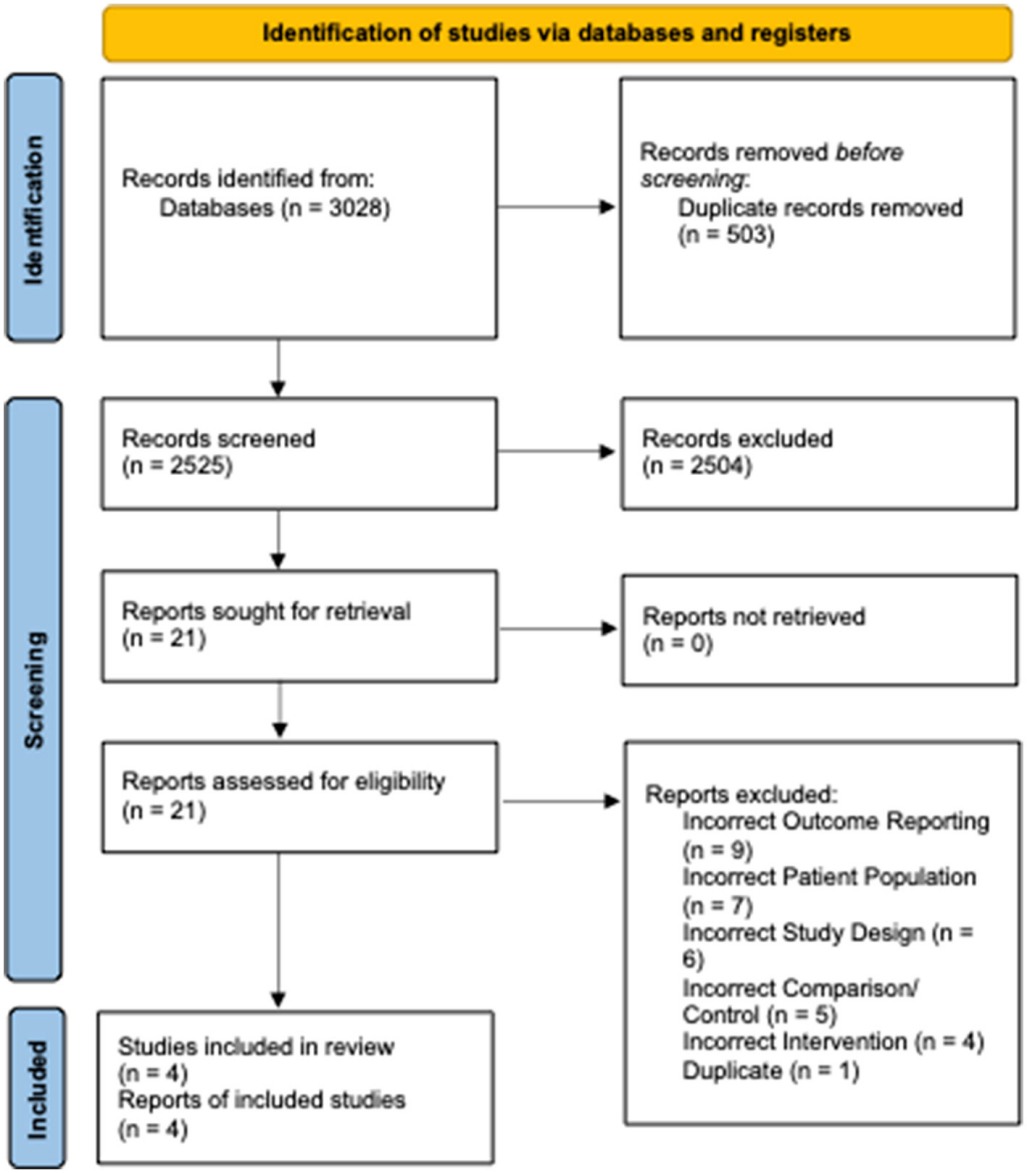
PRISMA diagram—transparent reporting of systematic reviews and meta-analysis flow diagram outlining the search strategy results from the initial search to included studies.

From 2,525 unique citations, four RCTs with 294 patients receiving preoperative VLEDs with liquid formulation (mean age: 40.8 years, female: 74.6%, and mean BMI: 44.1 kg/m^2^) and 294 patients receiving a non-VLED control (mean age: 41.4 years, female: 73.0%, and mean BMI: 44.1 kg/m^2^) were included (8, 12, 13, 19). A total of 21 studies were excluded at the full-text review stage (Appendix A1). A PRISMA flow diagram of the study screening process is presented in Figure 1. Included studies were conducted between 2011 and 2019. Postoperative morbidity was a secondary or tertiary outcome in all included studies. Study characteristics and demographic details for the included studies are presented in Table 1.

**Table 1:** Study characteristics of the included studies.

**References**	**Inclusion criteria**	**Exclusion criteria**	**Arms**	**N**	**Mean Age (SD)**	**% Female**	**Mean BMI (SD)**	**Comorbidities**	**Type of operation**
Van Nieuwenhove et al. (8)	Morbidly obese patients scheduled for laparoscopic gastric bypass at one of the participating centers; 18 to 60 years of age; previously attempted non-surgical programs for weight loss had failed; BMI greater than 40 or greater than 35 in the presence of obesity-related comorbidity	Patients who had undergone previous bariatric or gastric operations, those with severe psychological disorders, and those who could not be expected to adhere to the study protocol because of a language barrier or for any other reason	VLED	137	39.7 (9.5)	70.1	43.4 (10.0)	HTN−62 (45.3) T2DM−19 (13.9) OSA−21 (15.3) CVD−9 (6.6) Arthritis−27 (19.7)	RYGB−137 (100)
			Control	136	40.3 (9.7)	68.4	43.3 (8.2)	HTN−60 (44.1) T2DM−19 (14.0) OSA−15 (11.0) CVD−14 (10.3) Arthritis−23 (16.9)	RYGB−136 (100)
Schouten et al. (13)	Patients had to have a BMI >40 or >35 kg/m^2^ with at least two obesity-related comorbidities and suitable for gastric bypass surgery; undergoing gastric bypass or gastric sleeve surgery	Serious and/or untreated psychiatric disorders, serious cardiopulmonary disease with an ASA classification of 3 or higher, previous bariatric and/or gastric surgery including gastric banding and age <18 or >60 years	VLED	105	40.2	81	42.8	T2DM−38 (17.9) HTN−78 (36.8) DLD−55 (25.9) OSA−20 (9.4)	RYGB−99 (93.3) GS−6 (5.7)
			Control	107	41.7	76.6	43.1	Arthritis−137 (64.6)	RYGB−102 (95.3) GS−5 (4.7)
Contreras et al. (19)	Men and women who were morbid obese candidates for RYGB and GS bariatric surgery	A BMI of <35; Pregnant or breast-feeding women; Severe systemic or organ pathology; Insulin-treated; Have coagulation problems; Have unresolved eating disorders or severe psychiatric pathology	VLED	42	45.2 (10.5)	67.5	47.3 (5.3)	T2DM−11 (25.6) HTN−24 (55.8) OSA−11 (25.6) DLD−20 (46.5)	RYGB−28 (66.7) GS−14 (33.3)
			Control	41	45.3 (10.1)	75	47.3 (5.2)	T2DM−15 (17.9) HTN−35 (41.7) OSA−19 (22.6) DLD−35 (41.7)	RYGB−29 (70.7) GS−12 (29.3)
Chakravartty et al. (12)	All morbidly obese patients with a body mass index of >40 kg/m^2^ referred for weight loss surgery	Conditions where VLED use is contraindicated (e.g., severe hepatic impairment or portal hypertension, advancing renal impairment, pregnancy, lactation); Patients with independent factors for poor wound healing were excluded. Patients diagnosed with diabetes, smoking, preexisting chronic inflammatory disease, and using medications known to affect wound healing were not included; Patients with preexisting known skin conditions (e.g., psoriasis, eczema, and lupus) were also excluded.	VLED	10	43.5 (26–60)^*^	100	52.8 (42.1–63)^*^	HTN−3 (30) DLD−6 (60) OSA−4 (40) Arthritis−3 (30)	RYGB−10 (100)
			Control	10	38.5 (24–66)^*^	90	53.4 (45.1–61.7)	HTN−1 (10) DLD−3 (30) OSA−2 (20) Arthritis−3 (30)	RYGB−10 (100)

^*^Mean (range). SD, standard deviation; BMI, body mass index; VLED, very low energy diet; ASA, American Society of Anesthesiologists; T2DM, type II diabetes mellitus; HTN, hypertension; DLD, dyslipidemia; OSA, obstructive sleep apnea; CVD, cardiovascular disease; RYGB, roux-en-y gastric bypass; GS, gastric sleeve.

### Diet details

A detailed description of the preoperative VLEDs with liquid supplementation utilized for each of the included studies is shown in Table 2. Two of the studies utilized Optifast^Ⓡ^, one utilized Prodimed^Ⓡ^, and the other utilized a skim-milk liquid supplementation. All diets targeted 650–800 kcal per day. Duration of the preoperative VLED ranged from 2 to 4 weeks. Adherence was only reported by Contreras et al. they reported that 94% of patients included in the intervention arm consumed 80% or more of the prescribed doses of liquid supplementation (i.e., “high adherence”) (19).

**Table 2:** Preoperative very low energy diet details for individual included studies.

**References**	**N**	**Length of Diet (wks)**	**% Adherence**	**Liquid Formulation**	**Daily Macronutrients**	**Diet Details**	**Control Details**
Van Nieuwenhove et al. (8)	137	2	NR	Optifast^Ⓡ^	CHO: 100g Protein: 70g Fat: 15g	Target 800kcal/d 5 Optifast^Ⓡ^ shakes per day	Standard diet up to evening prior to surgery with Optifast^Ⓡ^ (no further details)
Schouten et al. (13)	105	2	NR	Prodimed^Ⓡ^	CHO: 12g Protein: 101g Fat: 16g	Target 650 kcal/d Liquid meal replacement plan	Target 657 kcal/d without Prodimed^Ⓡ^ CHO: 20g Fat: 25g Protein: 86g
Contreras et al. (19)	42	3	94	Optifast^Ⓡ^	CHO: 46.8% Protein: 36.4% Fat: 9.3%	Target 800 kcal/d 4 sachets of Optifast^Ⓡ^ per day Broth and non-calories beverages	Low-calorie diet 1200 kcal/d 2 sachets of Optifast^Ⓡ^ per day CHO: 46.8% Protein: 36.4% Fat: 9.3%
Chakravartty et al. (12)	10	4	NR	Semi-skimmed milk	CHO: 82g Protein: 61g Fat: 30g	Target 800 kcal/d 3 pints of semi-skimmed milk Each patient had multivitamin and mineral supplementation, further supplemented with a minimum of 2 L of energy-free liquid (water, diet fizzy drinks, mineral water, black tea/coffee, or squash with no added sugar) per day	NR

N, number of patients; wks, weeks; NR, not reported; CHO, carbohydrate; kcal, kilocalories; d, day; g, gram; L, liters.

### Control details

Specific diets were assigned to the control group in two of the RCTs. Gils-Contreras et al. randomly assigned patients 1:1 to VLED or low energy diet (LED). The LED had the same macronutrient composition (%) as the VLED; however, patients consumed approximately 1,200 kcal per day (vs. 800 kcal in the VLED group). Schouten et al. prescribed a strict “standard diet” to their control group that consisted of 657 kcal per day, 86 g of protein, 20 g of carbohydrate, and 25 g of fat. The control group in the RCT performed by Van Nieuwenhove et al. (8) did not alter the control group's diet. Patients were instructed to “have their regular diet” up to the evening prior to surgery. The most recent RCT by Chakravartty et al. (12) did not describe the control intervention.

### Preoperative weight loss

**Figure 2:**
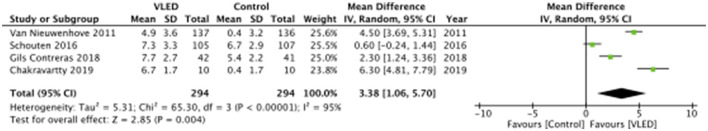
Preoperative weight loss—Random effects meta-analysis comparing VLED and control diets.

Anthropometric data for each included study are presented in Table 3. The mean pre-intervention weight loss for patients receiving VLEDs was 6.2 kg and for the patients receiving control was 3.4 kg. The mean reported weight change for all intervention groups across all studies was negative (i.e., on average, all patients, regardless of study arm lost weight). Upon pooling data from all four included studies, patients receiving VLED experienced significantly more weight loss than patients receiving control (MD 3.38 kg, 95%CI 1.06–5.70, *p* = 0.004, I^2^ = 95%) (Figure 2). The results were similar to the leave-one-out sensitivity analysis.

**Table 3:** Reported weight and body mass index outcomes for included studies.

**References**	**Arms**	**N**	**Mean pre-diet body weight (kg)**	**Mean post-diet body (kg)**	**Mean weight loss (kg)**	**Mean pre-diet BMI**	**Mean post-diet BMI**	**Mean BMI change**
Van Nieuwenhove et al. (8)	VLED	137	130.3 (23.7)	-	−4.9 (3.6)	43.4 (10.0)	-	−1.7 (1.3)
	Control	136	127.0 (22.8)	-	−0.4 (3.2)	43.3 (8.2)	-	−0.1 (1.1)
Schouten et al. (13)	VLED	105	122.5	115.2	−7.3	42.8	40.2	−2.6
	Control	107	125.0	118.3	−6.7	43.1	41.1	−2.0
Contreras et al. (19)	VLED	42	131.2 (22.4)	123.6 (21.0)	−7.7 (2.7)	47.2 (5.4)	44.5 (5.6)	−2.7 (0.8)
	Control	41	126.2 (17.1)	120.8 (16.0)	−5.4 (2.2)	47.2 (5.0)	45.2 (4.8)	−2.0 (0.8)
Chakravartty et al. (12)	VLED	10	125 (10–148)^*^	-	−6.7 (−4 to −9.4)^*^	53.4 (45.1–61.7)^*^	-	-
	Control	10	135.9 (110–15)^*^	-	−0.4 (−2.2 to 3.1)^*^	52.8 (42.1–63)^*^	-	-

^*^Mean (range). kg, kilograms; VLED, very low energy diet; N, number of patients; BMI, body mass index.

### Postoperative morbidity

**Figure 3:**
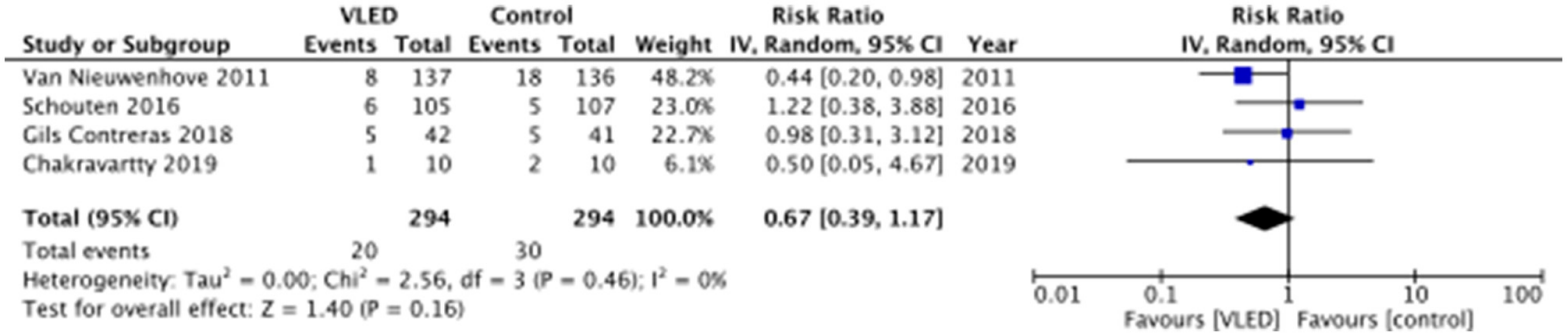
Postoperative morbidity—Random effects meta-analysis comparing VLED and control diets.

According to each of the included studies, postoperative morbidity is presented in Table 4. Three of the included studies reported 30-day postoperative morbidity as a composite of all system/organ-specific complications within 30 days of the index surgery, while Chakravartty et al. (12) reported 30-day postoperative morbidity without defining the types of complications (12). Upon pooling data from all four included studies, there was no significant difference in 30-day postoperative morbidity between patients receiving and not receiving VLED prior to bariatric surgery (RR 0.67, 95%CI 0.39–1.17, *p* = 0.16, I^2^ = 0%) (Figure 3). The results were similar to the leave-one-out sensitivity analysis.

**Table 4:** Reported perioperative outcomes for the included studies.

**References**	**Arms**	**N**	**Mean operative time, min (SD)**	**Mean intraoperative blood loss (mL)**	**% postoperative morbidity**	**Specific complications**
Van Nieuwenhove et al. (8)	VLED	137	80 (23)	30 (10–50)^**^	8 (5.8)	Wound−4 (2.9) GIB−1 (0.7) UTI−1 (0.7) PNA−1 (0.7) Other−1 (0.7)
	Control	136	81 (21)	30 (10–50)^**^	18 (13.2)	Wound−10 (7.4) AL−1 (0.7) GIB−1 (0.7) UTI−1 (0.7) PNA−2 (1.5) Other−3 (2.2)
Schouten et al. (13)	VLED	105	44	-	6 (5.7)	CVA−1 (0.9) Hemorrhage−1 (0.9) Anastomotic stenosis−1 (0.9) AKI−1 (0.9) Other−2 (1.8)
	Control	107	43	-	5 (4.8)	AKI−1 (0.9) UTI−2 (1.8) Other−2 (1.8)
Contreras et al. (19)	VLED	42	-	-	5 (11.9)	Minor−2 (4.8) Major−3 (7.1)
	Control	41	-	-	5 (12.2)	Minor−1 (2.4) Major−4 (9.8)
Chakravartty et al. (12)	VLED	10	139 (99–231)^*^	25 (10–50)^*^	2 (20.0)	-
	Control	10	129 (96–159)^*^	22.5 (20–100)^*^	1 (10.0)	-

^*^Mean (range); ^**^Median (range). N, number of patients; SD, standard deviation; mL, milliliters; VLED, very low energy diet; GIB, gastrointestinal bleed; UTI, urinary tract infection; PNA, pneumonia; AL, anastomotic leak; CVA, cerebrovascular accident; AKI, acute kidney injury.

### Risk of bias

**Figure 4:**
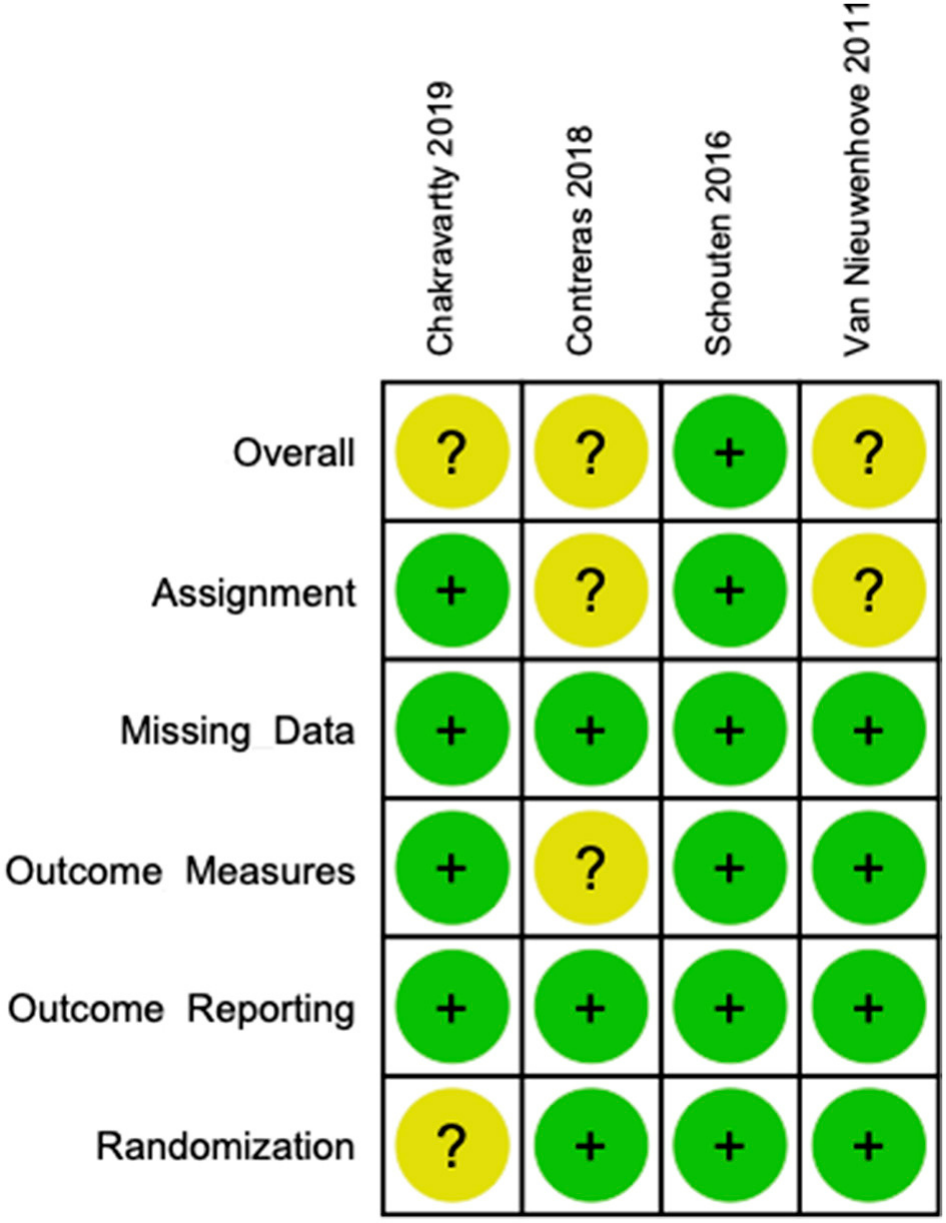
Cochrane risk of bias tool for randomized controlled trials 2.0—individual study analyses.

According to the Cochrane Tool, the risk of bias for RCTs 2.0 for each of the included studies is presented in Figure 4. The study by Schouten et al. was at low risk of bias across all domains (13). The studies by Van Nieuwenhove et al. and Contreras et al. were at unclear risk of bias due to deviations from the intervention without blinding of participants or the research team (8, 19). The study by Chakravartty et al. was at unclear risk of bias due to an imbalance of baseline covariates between the groups (12).

### Certainty of evidence

**Figure 5:**
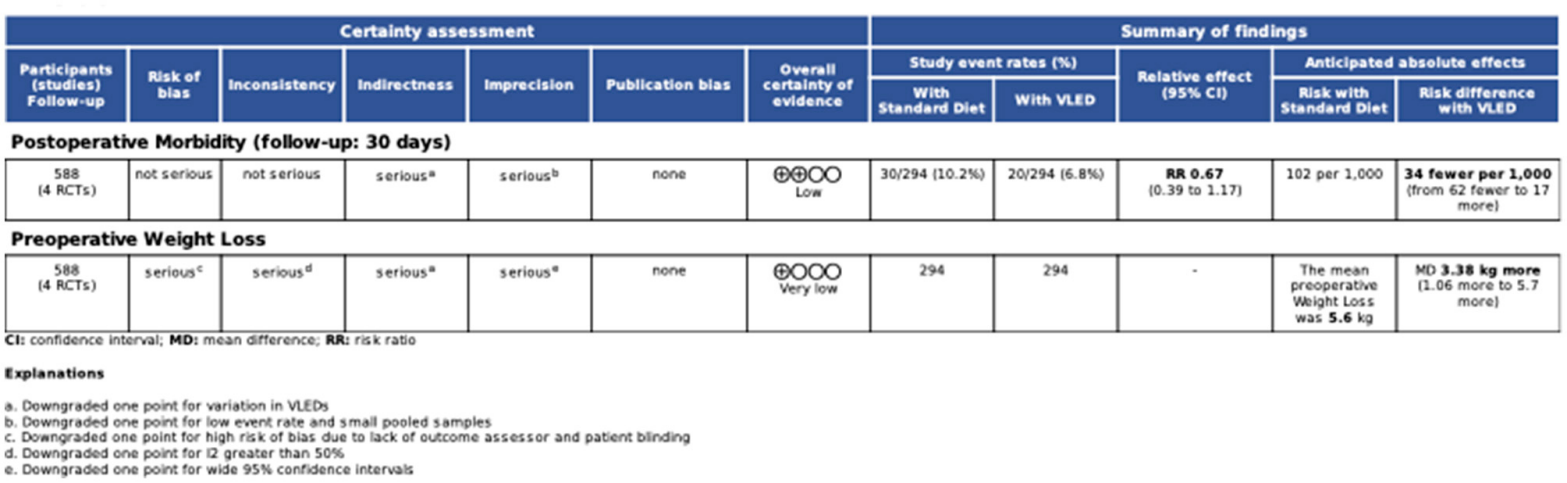
GRADE certainty of evidence summary table for meta-analyses.

The GRADE certainty of evidence summary table is presented in Figure 5. Overall, certainty of evidence for 30-day postoperative morbidity was low. The certainty of evidence supporting this outcome was downgraded due to heterogeneity in VLEDs, a small overall pooled sample size (*n* = 588), and a low outcome event rate (*n* = 50). Overall, certainty of evidence for preoperative weight loss was very low. The certainty of evidence supporting this outcome was downgraded due to heterogeneity in VLEDs, heterogeneity in the pooled estimate (i.e., I^2^ > 40%), wide 95% CIs for the pooled effect estimate, and high risk of bias due to lack of blinding.

## Discussion

While preoperative VLEDs for bariatric surgery are well established and are associated with benefits such as decreased visceral fat, surgeon-perceived difficulty, and operative time, data pertaining to their impact on postoperative morbidity are less robust (14, 15). This review identified four RCTs comparing VLEDs with liquid formulation to controls prior to bariatric surgery in terms of postoperative morbidity. Preoperative VLEDs with liquid formulation resulted in significantly more preoperative weight loss than control interventions. Overall, there was a point estimate suggesting a 33% reduction in the risk of overall 30-day postoperative morbidity with the use of VLEDs with liquid formulation—a point estimate suggesting an important benefit; however, the wide 95% CIs and resultant type II error risk create uncertainty (RR 0.67, 95% CI 0.39–1.17, *p* = 0.16). The certainty of evidence according to GRADE was low.

Obesity is associated with an increased risk of a number of postoperative complications across an array of surgical specialties (26). It induces systemic dysregulation across a number of biochemical pathways that, when combined with the physiologic stress of surgery, increase the vulnerability of the host to adverse events (27). In particular, obesity, which is often associated with insulin resistance, can have a major impact on surgical wound healing (8, 28). Not only there is heightened mechanical stress but also impaired cellular immune function due to insulin resistance and other obesity-associated comorbidities can increase the risk of surgical site infection, wound hematomas, wound seromas, and dehiscence (29, 30). Moreover, impaired immune function also places these patients at higher risk of postoperative infectious complications such as urinary tract infection and pneumonia (31). Obesity impacts the ability to mobilize in general, and this is exacerbated postoperatively, which could, in part, explain the increased rates of atelectasis and venous thromboembolism in these patients (32, 33). Obese patients are even at significantly greater risk of postoperative mortality compared with non-obese counterparts (31). Altogether, interventions targeted at reducing weight preoperatively for obese surgical patients should thus have the potential to impact postoperative outcomes. Moreover, preoperative VLEDs can improve glycemic control in patients with type II diabetes and thus may contribute to a reduction in postoperative complications by mitigating the adverse impacts of insulin resistance (34). Preoperative VLEDs in the present meta-analysis demonstrated an ability to reduce weight in a short period of time (mean weight loss of 6.2 kg). The meta-analysis of postoperative morbidity resulted in a point estimate suggesting an important benefit; however, the wide 95% CIs and resultant type II error risk create significant uncertainty as to whether the preoperative weight loss induced by the VLEDs significantly influenced postoperative morbidity (RR 0.67, 95%CI 0.39–1.17, *p* = 0.16, I^2^ = 0%).

Due to the systemic complications associated with obesity, as well as increased visceral and subcutaneous fat volume, obese patients across all surgical specialties, not just bariatric surgery, are at heightened postoperative risk (26, 35, 36). Thus, all obese surgical patients may also stand to benefit from preoperative VLEDs. Preoperative optimization via VLEDs for obese patients undergoing non-bariatric surgery has been studied with small RCTs and retrospective data. A systematic review by our research group published in 2022 identified 13 studies, evaluating their use prior to non-bariatric surgery (37). Postoperative morbidity was only reported in three RCTs, varying across orthopedic surgery and hepatobiliary surgery (35, 38, 39). However, similar to the present study, preoperative VLEDs were effective at inducing preoperative weight loss, with studies reporting preoperative weight loss ranging from 3.2 kg to 19.2 kg (37). The systematic review also noted consistent decreases in operative time and estimated blood loss, which can be associated with decreased postoperative morbidity (37). Along with the data from the present systematic review, we believe that preoperative VLEDs are safe for obese patients undergoing both bariatric and non-bariatric surgery that has the potential to decrease overall postoperative morbidity, but further study is required by way of large, high-quality RCTs.

The meta-analysis for overall 30-day postoperative morbidity in the present study suggested minimal between-study heterogeneity (I^2^ = 0%). However, there was significant heterogeneity in observed preoperative weight loss between studies (I^2^ = 95%). While there were insufficient data to explore heterogeneity through subgroup analyses, there were significant differences between the dietary interventions among the included studies that may explain the heterogeneity. Van Nieuwenhove et al. and Contreras et al. utilized Optifast™, Schouten et al. utilized Prodimed™, and Chakravartty et al. utilized a skim-milk liquid supplement (8, 12, 13, 19). Moreover, the duration of the intervention period varied across studies, with Van Nieuwenhove et al. and Schouten et al. intervening for 2 weeks, Contreras et al. intervening for 3 weeks, and Chakravartty et al. intervening for 4 weeks (8, 12, 13, 19). Both Optifast programs induced significant preoperative weight loss. The weight loss experienced by the patients in the intervention group compared with the control group in the Van Nieuwenhove et al. cohort was greater than the Contreras et al. cohort, despite the intervention period being a week shorter (4.5 kg vs. 2.3 kg) (8, 19). The only cohort that did not experience significant preoperative weight loss compared with control was that reported by Schouten et al., which utilized the Prodimed program (13). This program is much less commonly relied upon as a VLED than other programs such as Optifast, Modifast, and Formulite (15, 17, 40). The study that reported the largest MD in preoperative weight loss between intervention and control was Chakravartty et al., which also employed the longest intervention period (4 weeks). This may indicate that there is increased weight loss with longer intervention periods (12). Altogether, there remains significant heterogeneity in research and clinical practice in terms of VLED liquid formulation products and duration. Further study is required to determine the optimal intervention formulation and duration.

The strengths of this systematic review and meta-analysis include the thorough methodology, quality of the included studies, comprehensive risk of bias analysis, evaluation of the certainty of evidence with GRADE, and novelty. The study limitations include a small number of included studies (*n* = 4), small number of pooled participants (*n* = 588), small number of pooled outcome events (*n* = 50), lack of adherence data and heterogeneity of the included dietary interventions, and resultant weight loss. The small number of included studies and participants limited statistical power, such that the meta-analysis was underpowered to detect a difference in overall 30-day postoperative morbidity. Heterogeneity in VLED interventions, specifically the type of liquid supplementation and duration of the intervention, may impact compliance, weight loss outcomes, and postoperative outcomes (19, 40). Similarly, significance between study heterogeneity existed in terms of control interventions, with some studies including active controls and others not, thus potentially attenuating the relative risk reduction observed with the intervention. These effects could not be explored via subgroups due to the deficit in the quantity of included data. The data from the included studies were limited due to a high risk of bias, mostly due to a lack of blinding and a paucity of compliance data.

In summary, the impact of preoperative VLEDs on postoperative outcomes following bariatric surgery remains unclear. It is possible that VLEDs may contribute to decreased postoperative morbidity, but further larger prospective trials are required to investigate the signal identified in this study. Furthermore, large trials investigating the use of preoperative VLEDs in obese patients undergoing non-bariatric surgery are required to determine the generalizability of these interventions aimed toward optimizing a continuously growing patient population.

## Author contributions

All authors involved in the conception and design of the study, acquisition of data, analysis and interpretation of data, drafting and revision of the manuscript, approval of the final version of the manuscript, and agreement to be accountable for all aspects of the study.

## Appendix

**Table A1:** Reason for exclusion of studies during full text screen (excluding duplicates).

**Study**	**Reason for exclusion**
Alami et al. (41)	Did not compare weight loss interventions, just whether weight loss occurred or not
Faria et al. (18)	Compared two different types of VLEDs
Carbajo et al. (10)	Did not report postoperative morbidity
Bakker et al. (11)	Did not report postoperative morbidity
Baldry et al. (42)	Did not report postoperative morbidity
Nielsen et al. (43)	Compared two different durations of VLEDs
Kalarchian et al. (44)	Assessed complex lifestyle intervention, not including VLED
Heinberg et al. (9)	Did not report postoperative morbidity
Lorenzo et al. (45)	Non-randomized study design
Kandel et al. (46)	Patients had previously undergone bariatric surgery, were no longer in the preoperative period
Albanese et al. (47)	Non-randomized study design
Tauser et al. (48)	Patients had previously undergone bariatric surgery, were no longer in the preoperative period
Hutcheon et al. (49)	Non-randomized study design
Parikh et al. (50)	Assessed complex lifestyle intervention, not including VLED
Davenport et al. (40)	Compared two different types of VLEDs
Crujeiras et al. (51)	Non-randomized study design
Jones et al. (52)	Non-randomized study design
