# Supplementary material for: Very low energy diets prior to bariatric surgery may reduce postoperative morbidity: a systematic review and meta-analysis of randomized controlled trials
Source: Front Nutr. 2023 Jun 20;10:1211575. doi: 10.3389/fnut.2023.1211575 (PMC10319356; doi:10.3389/fnut.2023.1211575)
Supplement: Supplementary file 1 [file Data_Sheet_1.docx]

| 1. Bariatric Surgery/ 2. Gastric Bypass/ 3. Gastroplasty/ 4. Jejunoileal Bypass/ 5. Gastric Sleeve.mp. 6. Sleeve Gastroplasty.mp. 7. Vertical Sleeve Gastroplasty.mp. 8. Roux-en-y Gastric Bypass.mp. 9. Duodenal Switch.mp. 10. Single Anastomosis Duodenal Interposition.mp. 11. Biliopancreatic Diversion/ 12. Anastomosis, Roux-en-Y/ 13. Gastric Banding.mp. 14. Very Low Energy Diet.mp. 15. Caloric Restriction/ 16. Diet, High-Protein Low-Carbohydrate/ 17. Diet, Fat-Restricted/ 18. Diet, Reducing/ 19. Very Low Calorie Diet.mp. 20. Weight Loss Program*.mp. 21. Weight Loss Intervention*.mp. 22. Weight Loss Therapy.mp. 23. Total Meal Replacement.mp. 24. Optifast.mp. 25. Optislim.mp. 26. Modifast.mp. 27. Obecure.mp. 28. Or/1-13 29. Or/14-27 30. 28 and 29 31. Animals 32. Humans/ 33. 31 not (31 and 31) 34. 30 not 33 |
| --- |

**Appendix 1.** Complete search strategy (Medline and Embase database example)
