# Supplementary material for: Very low energy diets prior to bariatric surgery may reduce postoperative morbidity: a systematic review and meta-analysis of randomized controlled trials
Source: Front Nutr. 2023 Jun 20;10:1211575. doi: 10.3389/fnut.2023.1211575 (PMC10319356; doi:10.3389/fnut.2023.1211575)
Supplement: Supplementary file 2 [file Data_Sheet_2.docx]

| (“bariatric surgery”):ti,ab,kw OR (“gastric bypass surgery”):ti,ab,kw OR (“gastric sleeve”):ti,ab,kw (word variations have been searched)  AND  (“very low energy diet”):ti,ab,kw OR (“very low calorie diet”):ti,ab,kw (word variations have been searched) |
| --- |

**Appendix 2.** Complete search strategy (CENTRAL database example)
